# Biphasic Effect of Sildenafil on Energy Sensing is Mediated by Phosphodiesterases 2 and 3 in Adipocytes and Hepatocytes

**DOI:** 10.3390/ijms20122992

**Published:** 2019-06-19

**Authors:** Jheelam Banerjee, Antje Bruckbauer, Teresa Thorpe, Michael B. Zemel

**Affiliations:** NuSirt Biopharma Inc., 11020 Solway School Rd, Knoxville, TN 37931, USA; banerjee.jheelam@gmail.com (J.B.); Mrlnnsbhn671@gmail.com (T.T.); mzmemel@nusirt.com (M.B.Z.)

**Keywords:** AMPK, Sirt1, PDE2, PDE3, PDE5, leucine, sildenafil, NASH, obesity

## Abstract

Sirt1 (Sirtuin 1), AMPK (AMP-activated protein kinase), and eNOS (endothelial nitric oxide synthase) modulate hepatic energy metabolism and inflammation and play a major role in the development of NASH. Cyclic nucleotide phosphodiesterases (PDEs) play an important role in signal transduction by modulating intracellular levels of cyclic nucleotides. We previously found the PDE5 inhibitor sildenafil to synergize with leucine and leucine-metformin combinations in preclinical studies of NASH and obesity. However, efficacy is diminished at higher sildenafil concentrations. Herein, we have successfully modeled the U-shaped sildenafil dose-response in vitro and utilized this model to assess potential mechanisms of this dose-response relationship. Adipocytes and liver cells were treated with leucine (0.5 mM) and different concentrations of sildenafil (1 nM to 100 µM). cAMP, cGMP, and P-AMPK protein expression were used to demonstrate the biphasic response for increasing concentrations of sildenafil. The reversal with higher sildenafil levels was blunted by PDE2 inhibition. These data indicate that sildenafil-mediated increases in cGMP inhibits PDE3 at lower concentrations, which increases cAMP. However, further increases in cGMP from higher sildenafil concentrations activate PDE2 and consequently decrease cAMP, which demonstrates crosstalk between cAMP and cGMP via PDE2, PDE3, and PDE5. These changes in cAMP concentration are further reflected in downstream effects, including AMPK activation.

## 1. Introduction

Nonalcoholic steatohepatitis (NASH) is a complex disease involving multiple molecular pathways as well as numerous genetic, epigenetic, and environmental contributors [1,2]. Sirt1 and AMPK are important nutrient sensors activated by AMP and NAD^+^, respectively, that coordinately regulate energy metabolism including hepatic glucose and lipid metabolism [3,4,5]. The endothelial nitric oxide synthase, nitric oxide, and cyclic guanosine monophosphate (eNOS-NO-cGMP) signaling pathway interacts bi-directionally with this network, as eNOS is phosphorylated and acetylated by AMPK and Sirt1, respectively, while nitric oxide stimulates SIRT1 activity [6,7]. Consequently, targeting this network from multiple sites leads to synergistic stimulation of downstream effects. We have previously demonstrated that L-Leucine, which is an allosteric Sirt1 activator, synergizes with low doses of metformin, an AMPK activator, and sildenafil, which is a well-known PDE 5 inhibitor, to modulate hepatic lipid metabolism and reverse NASH in preclinical mouse models [8,9,10,11].

Cyclic nucleotide phosphodiesterases (PDEs) play an important role in signal transduction by modulating intracellular levels of cyclic nucleotides [12]. By catalyzing the hydrolysis of adenosine 3′, 5′-cyclic monophosphate (cAMP) and guanosine 3′,5′-cyclic monophosphate (cGMP), PDEs lower the intracellular concentrations of cyclic nucleotides cAMP and cGMP to their respective 5′ nucleoside monophosphates. The 11 known PDE families include at least 60 species of PDEs, which differ in their substrate affinity, allosteric regulation, and tissue distribution [13]. While PDE5 is specific for hydrolysis of cGMP, PDE2, and PDE3 are both able to use cGMP and cAMP as substrates with different affinities [14]. Moreover, cGMP enhances cAMP levels by competing for the active site of PDE3. However, at higher cGMP concentrations, cGMP also acts as an allosteric activator for PDE2 and, thereby, increases cAMP hydrolysis, which provides crosstalk between cGMP and cAMP signaling pathways [15,16].

Sildenafil’s predominant action is the inhibition of phosphodiesterase 5 (PDE5) and a resultant increase in intracellular cGMP, which results in therapeutic utility for both erectile dysfunction and pulmonary hypertension [17,18,19]. However, low concentrations of sildenafil also activate endothelial nitric oxide synthase (eNOS), which results in nitric oxide induced increase in cGMP as well as in stimulation of the AMPK/Sirt1/PGC1α pathway and synergy with leucine [18,20,21]. Furthermore, the cGMP-cAMP feedback system may either amplify or inhibit the effects of Sild-Leu on the target AMPK/Sirt1/PGC1α pathway, since changes in cAMP are also reflected in corresponding changes in AMPK activity via the PKA-EPAC1 pathway [22].

Cyclic nucleotide-regulated signal transduction can be initiated through several mechanisms, i.e., by cyclic nucleotide-induced activation of cAMP-protein and cGMP-protein kinases (PKA and PKG, respectively), with subsequent phosphorylation and regulation of downstream effectors, or by binding to and activation of specific cyclic nucleotide binding proteins, which directly mediate cyclic nucleotide actions [23,24,25]. The EPAC proteins EPAC1 and EPAC2 are cAMP-activated guanine-nucleotide-exchange proteins, which regulate several pivotal cellular processes including cell proliferation, cell survival, cell differentiation, gene transcription, and ion transport [26]. EPAC mediated AMPK activation involves the increase of intracellular Ca^2+^ and activation of the calcium/calmodulin-dependent kinase kinase β (CamKKβ), which presents a PKA-independent downstream effect of cAMP signaling [27,28,29,30].

We previously found sildenafil to synergize with leucine and leucine-metformin combinations in preclinical studies of NASH and obesity and that a leucine-metformin-sildenafil combination exerted a therapeutic benefit in nonalcoholic fatty liver disease (NAFLD) and obesity [9,31,32,33]. However, we observed a sildenafil “U-shaped” dose response relationship for most NASH-related variables and body weight, with diminished efficacy at higher sildenafil concentrations (unpublished data). Herein, we have successfully modeled the U-shaped sildenafil dose-response in vitro and utilized this model to assess potential mechanisms of this dose-response relationship. Our data demonstrate an expected cGMP response to sildenafil, which is accompanied by a biphasic cAMP response. We hypothesize that, at low concentrations, sildenafil can inhibit PDE3 and, thereby, induce up to a two-fold increase in cAMP, whereas, at higher concentrations, this effect is reversed due to indirect activation of PDE2. These effects on cAMP are subsequently reflected in changes in AMPK phosphorylation and activation.

## 2. Results

### 2.1. The Biphasic Response of cAMP to Increasing Sildenafil Concentrations is Mediated by PDE2

**Figure 1:**
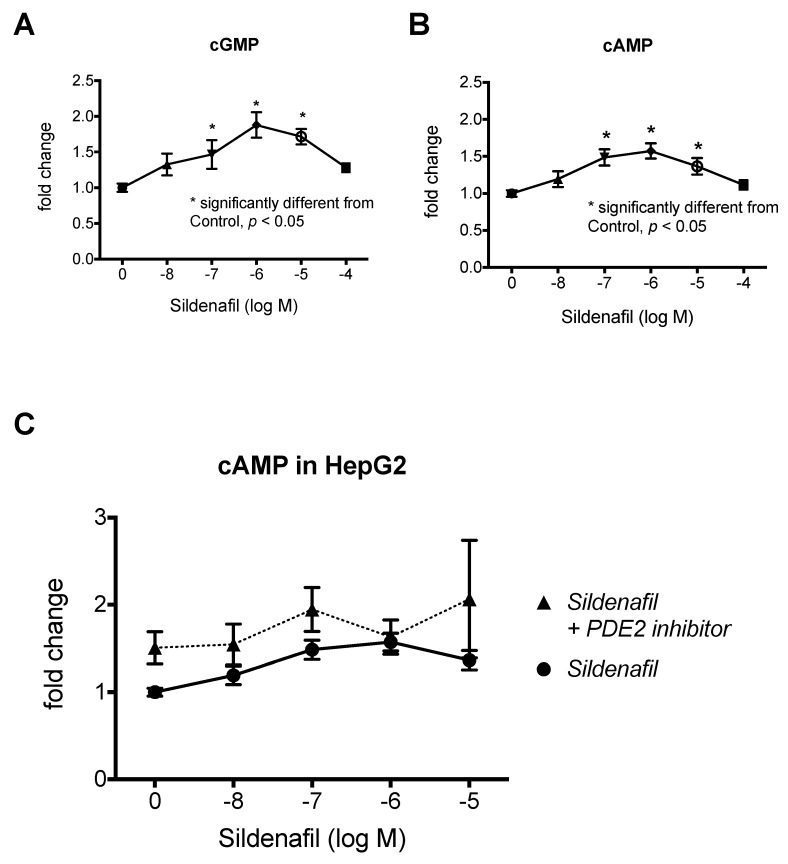
Effects of sildenafil on cGMP and cAMP concentrations in HepG2 cells. (**A**) Intracellular cGMP and (**B**) intracellular cAMP concentrations in HepG2 cells treated with sildenafil (10 nM to 100 µM) for 30 min. Data were calculated and statistically analyzed as fold change of control and are expressed as means ± SEM (*n* = 3 to 6, *p* ≤ 0.05) (absolute values for control means are 1.642 pmol cGMP/mg protein and 3.45 pmol cAMP/mg protein). (**C**) Comparison of cAMP response in HepG2 cells treated with sildenafil (10 nM to 10 µM) +/− PDE2 inhibitor (BAY 60-7550, 10 uM) for 30 min.

**Figure 2:**
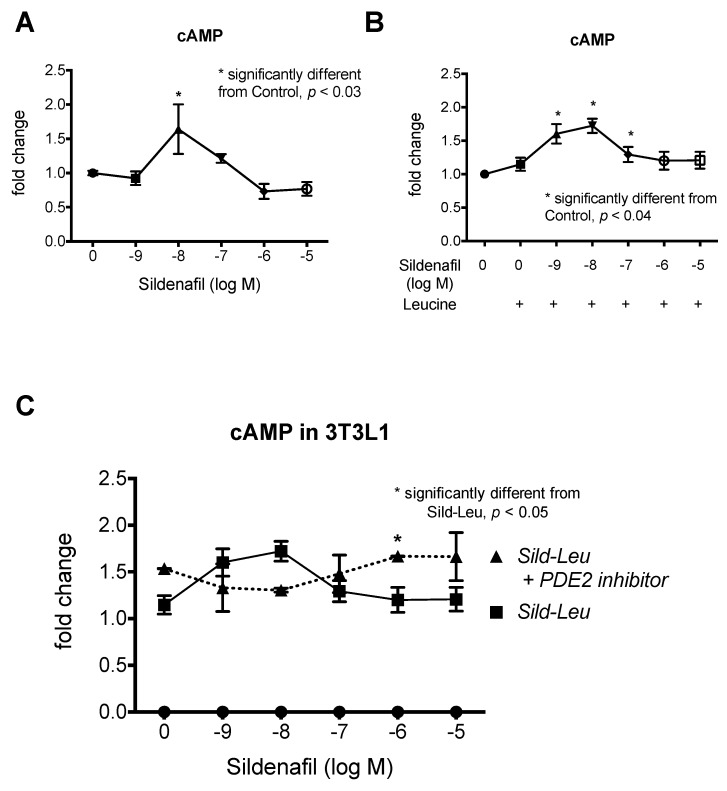
Effects of sildenafil and leucine on cAMP concentrations in 3T3L1 cells. cAMP response in 3T3L1 adipocytes treated with (**A**) sildenafil (1 nM to 10 µM) alone or (**B**) in combination with Leucine (0.5 mM) for 30 min. Data were calculated and statistically analyzed as a fold change of control and are expressed as means ± SEM (*n*= 3 to 5, *p* ≤ 0.05) (absolute values for control means are 3.922 and 3.48 pmol cAMP/mg protein for Sildenafil and Sildenafil-Leucine, respectively). * indicates a significant difference from Control (*p* < 0.04). (**C**) Comparison of cAMP response in 3T3L1 adipocytes treated with sildenafil-leucine +/− PDE2 inhibitor (BAY 60-7550, 10 µM) for 30 min. * indicates significant difference between the two curves (*p* < 0.05).

To model the biphasic dose response of sildenafil in vitro, we first tested the effects of sildenafil on cGMP and cAMP concentrations in HepG2 hepatocytes. As expected, increasing concentrations of sildenafil produced a corresponding increase in cGMP up to 1 µM and a decrease with 10 and 100 µM (Figure 1A). Similarly, we observed a biphasic response in cAMP with increasing concentrations up to 1 µM sildenafil treatment and a decrease with 10 and 100 µM sildenafil treatment (Figure 1B). We confirmed the biphasic effects of sildenafil in 3T3L1 adipocytes (Figure 2A). Similar effects were also observed with increasing concentrations of sildenafil in combination with a fixed concentration of leucine (0.5 mM), which demonstrated that the addition of leucine does not contribute to this biphasic response (Figure 2B). The addition of the PDE2 inhibitor BAY 60-7550 prevented the decrease of cAMP at a high level of sildenafil (1 and 10 µM) concentration in combination with leucine in adipocytes (Figure 2C), while there was only a non-significant trend in hepatocytes (Figure 1C).

### 2.2. The Biphasic cAMP Response to Sildenafil is Reflected in Downstream Effects

**Figure 3:**
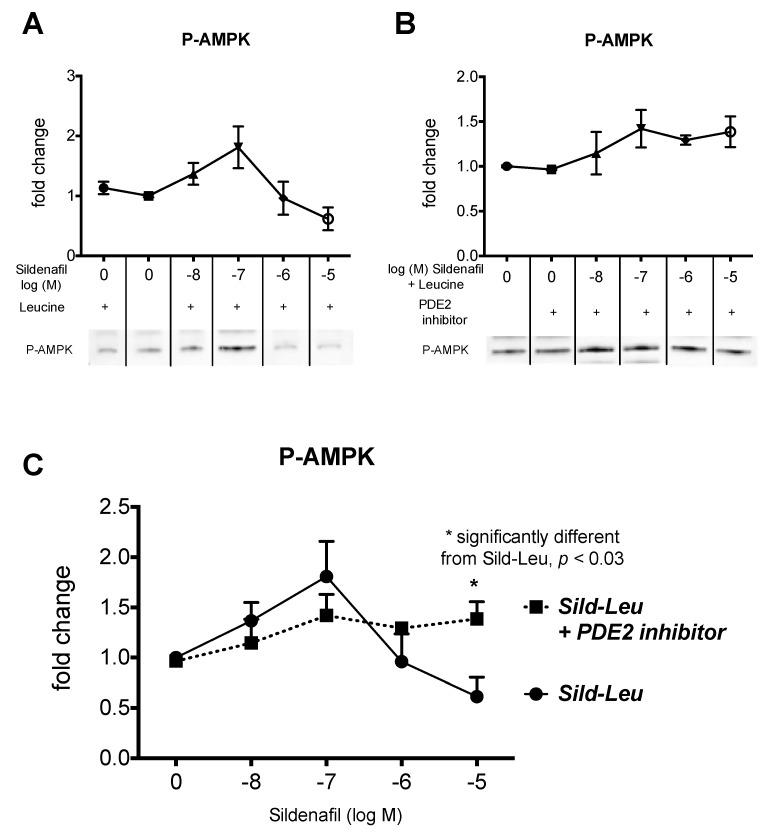
Protein expression of Phospho (Thr172)-AMPK in HepG2 cells. Protein expression of Phospho (Thr172)-AMPK in HepG2 cells treated with a fixed dose of leucine (0.5 mM) in combination with (**A**) sildenafil (10 nM to 10 µM) or (**B**) sildenafil (10 nM to 10 µM) plus the PDE2 Inhibitor (BAY 60-7550, 10 uM) for 24 h. Graphs were calculated from pooled densitometry measurements of two different blots (*n* = 3 to 4) and representative blots are shown. (**C**) Comparison of P-AMPK response to Sildenafil-Leucine for the addition of the PDE2 inhibitor. * indicates a significant difference between the curves (*p* < 0.03).

**Figure 4:**
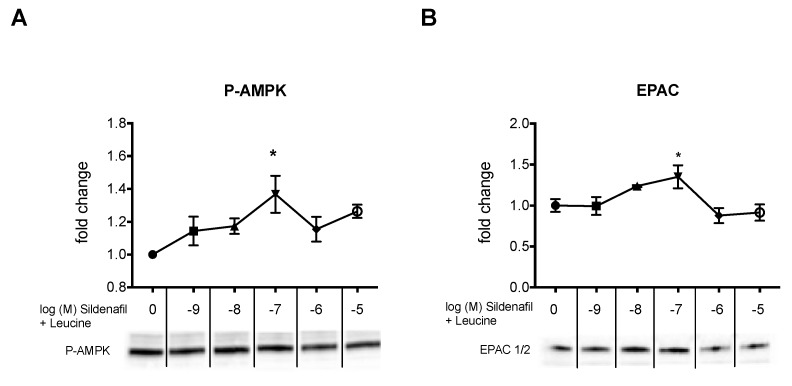
Protein expression of Phospho (Thr172)-AMPK and EPAC in 3T3L1 adipocytes. (**A**) P-AMPK and (**B**) EPAC protein expression in 3T3L1 cells treated with sildenafil (1 nM to 10 µM) in combination with leucine (0.5 mM) for 2 hours. Graphs were calculated from pooled densitometry measurements of two different blots (*n* = 4) and representative blots are shown. * indicates a significant difference from the control (*p* < 0.05).

Next, we tested whether the biphasic response of cAMP was reflected in similar activation of the AMPK pathway in HepG2 hepatocytes. A significant upregulation of *p*-AMPK was observed at an increasing concentration of sildenafil-leucine, which peaked at 100 nM sildenafil and then decreased (Figure 3A). This biphasic response was inhibited by the addition of PDE2 inhibitor (Figure 3B). There was no significant change in unphosphorylated AMPK. We repeated the results on P-AMPK in 3T3L1 adipocytes (Figure 4A) and also observed a similar response curve to increasing sildenafil concentration on the EPAC1 protein expression (Figure 4B), which suggests that the effects of cAMP on P-AMPK are mediated by EPAC1 as a downstream effector of cAMP. 

### 2.3. Knockdown of PDE2 Prevents the Biphasic Sildenafil Response of cAMP and Downstream Targets

**Figure 5:**
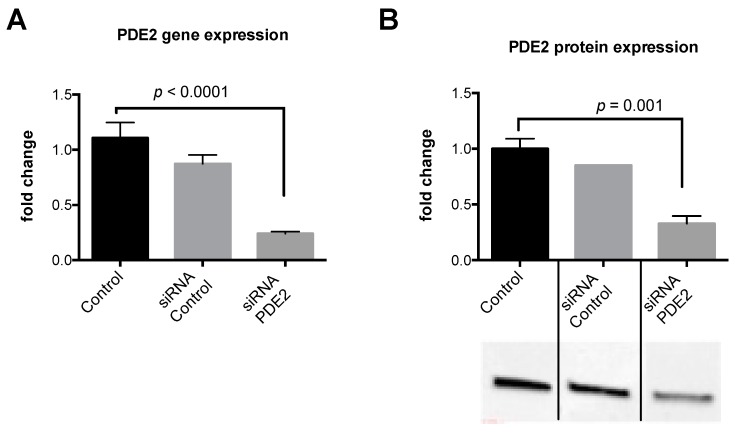
Effect of PDE2 knockdown on PDE2 gene and protein expression. HepG2 cells were transfected with siRNA against PDE2 for 48 hours. (**A**) Gene expression of PDE2 and (**B**) protein expression were measured to confirm the silencing. Bar graphs of densitometry measurements and representative blots are shown (*n* = 3).

**Figure 6:**
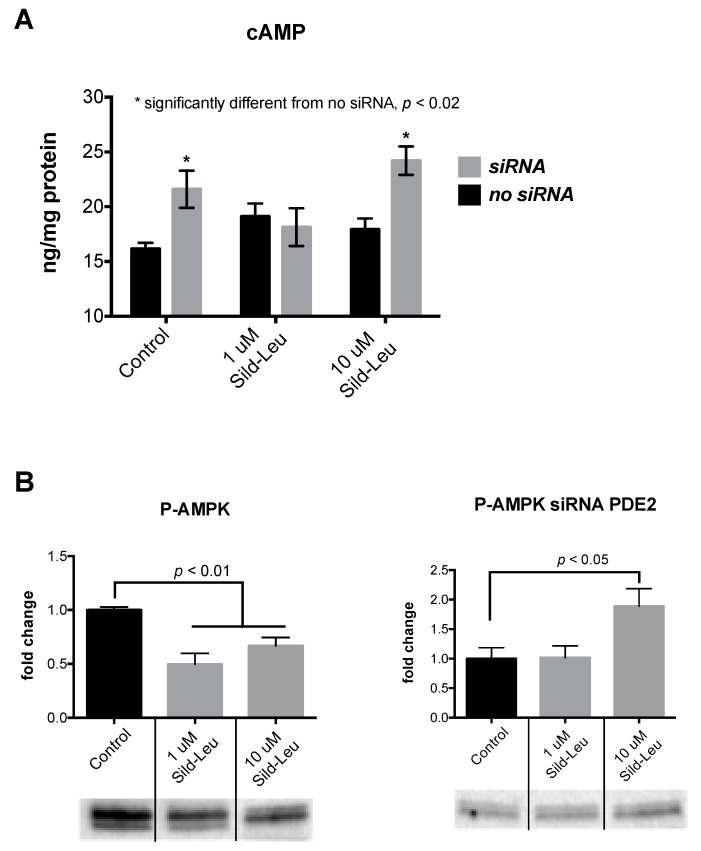
Change in cAMP and P-AMPK protein expression for treatment with PDE siRNA. HepG2 cells were treated with or without PDE2 siRNA for 48 hours, which was followed by 30-minute treatment with Sildenafil (1 or 10 µM) – Leucine (0.5 mM). (**A**) Intracellular cAMP concentration was measured. * indicates a significant difference between the cells treated with siRNA as opposed to the ones not treated with PDE2 siRNA (*p* < 0.02). (**B**) Phospho (Thr172)-AMPK protein expression was measured. Bar graphs of densitometry measurements and representative blots are shown (*n* = 3).

We then performed siRNA experiments to silence PDE2 in HepG2 hepatocytes. We confirmed the silencing via both PCR and protein expression. PDE2 expression was reduced by ~70% after treating the cells with PDE2 siRNA (Figure 5). After silencing, we treated the cells with sildenafil (1 uM and 10 µM) in combination with leucine. Similar to the results with the PDE2 inhibitor, cAMP concentrations were increased at 10 uM Sild-Leu in PDE2 knockdown cells compared to the wild-type cells (Figure 6A), and the reduction in P-AMPK expression with the higher Sild-Leu dose (10 µM) was prevented in the knockdown cells (Figure 6B).

## 3. Discussion

The main finding of this paper is that, at low concentrations, sildenafil induces up to a two-fold increase in cAMP, which is, most likely, caused by inhibition of PDE3, whereas, at higher concentrations, this effect is reversed due to cGMP induced activation of PDE2. These effects on cAMP are reflected in corresponding changes in the AMPK activation (P-AMPK).

Our previous preclinical and clinical studies showed a biphasic dose response curve for Sildenafil in combination with a fixed dose of Leucine or Metformin-Leucine with respect to both body fat and NASH-related liver outcomes. Therefore, we focused our cell studies modeling these effects on hepatocytes and adipocytes. Both cell types showed similar responses in cAMP for increasing sildenafil concentrations. However, the peak was at 10 nM sildenafil in adipocytes and at 100 nM in hepatocytes.

It was previously reported that treatment with low concentrations of sildenafil plus sodium nitroprusside (SNP, 1uM) increased intracellular cAMP, possibly indirectly via cGMP inhibition of PDE3. At higher concentrations of sildenafil, cAMP levels decreased concentration-dependently, possibly through the reported activation of PDE2 caused by higher intracellular cGMP concentrations [34]. Although cAMP and cGMP-signaling can control cellular events with remarkable selectivity, these systems do not always act in isolation [35]. Studies have previously shown that, while activators of nitric oxide sensitive guanylyl cyclases increased levels of cGMP, they also regulated levels of platelet cAMP [36].

Leucine synergizes with sildenafil and other eNOS activators to exert amplifying downstream effects of AMPK and Sirt1 activation on glucose and fat metabolism [9,11]. When we tested this hypothesis with a fixed dose of leucine and increasing concentrations of sildenafil, we found a significant upregulation of P-AMPK up to 100 nM sildenafil followed by decreases at higher doses (Figure 1). Although the best known action of sildenafil is inhibition of PDE5, sildenafil also activates eNOS, which results in increased NO/cGMP signaling with subsequent activation of the cGMP-dependent protein kinases (PKGs) [37,38]. Moreover, leucine synergizes with sildenafil to amplify downstream effects on AMPK, and pathway to reverse NAFLD in preclinical mouse models [9].

**Figure 7:**
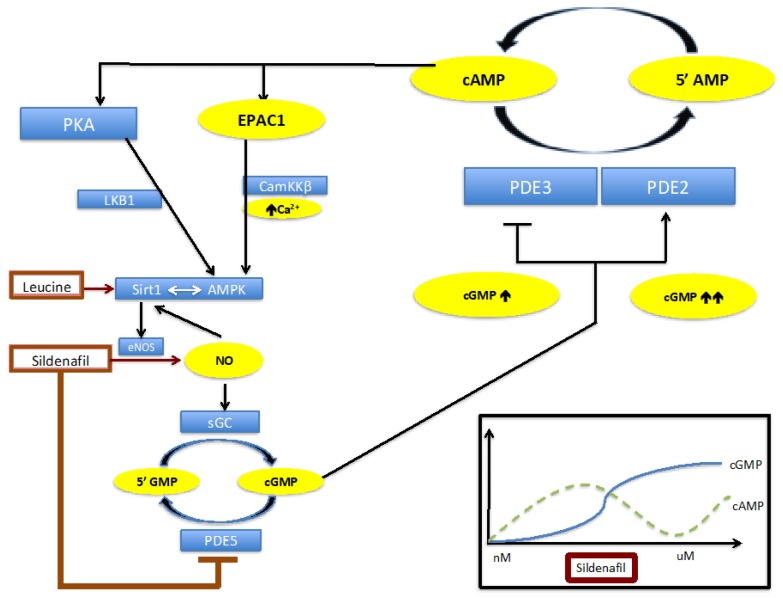
Schematic representation of the interaction between Sildenafil, Leucine, PDE2, and PDE3. Increasing concentrations of sildenafil cause an increase in intracellular cGMP due to eNOS/NO activation and PDE5 inhibition, which is accompanied by a biphasic response in cAMP (lower right corner). This biphasic response in cAMP is mediated by the interaction of cGMP with PDE2 and PDE3. While PDE3 is inhibited by moderate increases in cGMP and consequently cAMP concentrations rise due to reduced hydrolysis, higher levels of cGMP activate PDE2 and, therefore, decrease cAMP. These changes in cAMP are reflected in downstream events such as AMPK activation, which is most likely mediated over the cAMP-EPAC-CamKKβ signaling pathway.

To address the role of PDE2 on the observed biphasic response to sildenafil, we inhibited PDE2 by both silencing and by chemical inhibition (BAY 60-7550). We found the reduction in cAMP and AMPK activation at higher sildenafil concentrations was prevented with PDE2 suppression (Figure 2 and Figure 3). As summarized in Figure 7, our data suggest that the increase in cAMP at low sildenafil concentrations is caused by cGMP-mediated inhibition of PDE3. However, at higher sildenafil concentrations, the rising cGMP concentration leads to activation of PDE2, which leads to cAMP degradation. Further increases in cGMP activate PDE2, which reduces cAMP. This leads to a reduction in AMPK activation. These results also document that a cross talk between PDE2 and PDE5 operates, which strengthens the physiological relevance of these findings.

This interplay between cGMP and cAMP mediated by PDE2-PDE3-PDE5 is supported by previous studies. Isidori et al. tested the beta-adrenergic (bAR) contraction rate, which is reflected by a cAMP increase, in response to sildenafil in isolated neonatal cardiomyocytes [39]. Inhibition of PDE5 with 1 µM sildenafil significantly lowered the bAR-stimulated contraction rate, which was associated with increased cGMP levels. This effect was abolished by the addition of a specific PDE2 inhibitor EHNA, and was further confirmed in heterozygous PDE2 knockout mice, indicating the indirect activation of PDE2 by sildenafil, and, consequently, increased cAMP hydrolysis. This is responsible for the effects of PDE5 inhibition on the bAR contraction rate.

Our data support an EPAC-mediated mechanism, as EPAC protein expression showed the same biphasic response as the cAMP concentration induced by increasing concentration of sildenafil (Figure 4B). While EPAC1 is ubiquitously expressed in all tissues, EPAC2 is more limited in its tissue distribution, and they are involved in a host of cAMP-related cellular functions [40]. Park et al. reported that there is a competitive inhibition of cAMP-degrading phosphodiesterases, which leads to elevated cAMP levels, results in the activation of EPAC1, and subsequent activation of AMPK [22]. In addition, others have suggested that both cAMP effectors EPAC and PKA may directly activate eNOS via Ser 1177 phosphorylation by activating the PI3K/Akt pathway, which may further contribute to the activation of the AMPK-Sirt1-eNOS network [41].

## 4. Materials and Methods

### 4.1. Cell Culture

HepG2 cells (ATCC) were either grown in low glucose DMEM (5 mM glucose) or high glucose DMEM (25 mM glucose) containing 10% fetal bovine serum (FBS) and antibiotics (1% penicillin-streptomycin) at 37 °C in 5% CO_2_ in air. 

Murine 3T3-L1 pre-adipocytes (ATCC) were grown in the absence of insulin in Dulbecco’s modified Eagle’s medium (DMEM, 25 mM glucose) containing 10% fetal bovine serum (FBS) and antibiotics (1% penicillin-streptomycin) (adipocyte medium) at 37 °C in 5% CO_2_ in air. Confluent pre-adipocytes were induced to differentiate with a standard differentiation medium (DM2-L1, Zen-Bio Inc., Research Triangle Park, NC, USA). Pre-adipocytes were maintained in this differentiation medium for 3 days and, subsequently, cultured in adipocyte medium for 8 to 10 days more to allow 90% of cells to reach full differentiation before treatment. The medium was changed every 2 to 3 days. Differentiation was determined microscopically through the inclusion of fat droplets.

### 4.2. siRNA Transfection

HepG2 cells were seeded with 10^4^ cells/well on a 96-well plate or 6 well plates (confluence, ~50 to 60%) in antibiotic-free medium containing the siRNA to be transfected to the cells. The cells were transfected with siRNA against PDE2 (Ambion, ID#143736) complexed to a Lipofectamine RNAiMAX reagent (ThermoFisher Scientific, Cat# 13778-030), according to the manufacturer’s instructions for 48 h. Media was replaced to medium containing antibiotics for 48 h. Cells were harvested using the cells to CT method and RT was performed. PCR for PDE2 with 18 s as the reference gene was performed to determine the extent of the silencing in the cells. Western blots were also performed on the cells harvested from the 6 well plates.

### 4.3. Gene Expression

Cells were grown in a 96-well plate. Cell Lysis, reverse transcription, and RT-PCR were performed using the TaqMan^®^ Gene Expression Cells-to Ct™ Kit (Life Technologies (Thermo Fisher Scientific, Waltham, MA, USA), Cat # 4399002), according to the manufacturer’s instructions. Gene expression was assessed by RT-PCR using the StepOnePlus™ PCR system (Thermo Fisher Scientific, Waltham, MA, USA) and TaqMan Gene expression assays for PDE2 (Life Technologies, Cat #HS00159935_m1)).

### 4.4. cAMP and cGMP Assay

HepG2 and 3T3L1 cells were seeded in T75 flasks in high glucose DMEM. After 48 h, they were treated with various concentrations of Sild (1 nM, 10 nM, 100 nM, 1 uM, 10 uM, and 100 uM) with or without a fixed concentration of Leucine (0.5 mM) and the PDE2 inhibitor (BAY 60-7550, 10 uM) for 30 min. Then cells were washed in PBS and incubated for 20 min in 0.1 M HCl. The cells were harvested and spun at 600 g for 10 min. The supernatant was collected and the same was used for both the cGMP Assay (Abcam, Cambridge, MA, USA ab133027) and the cAMP assay (Enzo Life Sciences, Farmingdale, NY, USA, Cat# ADI-900-066) that were run according to the manufacturer’s protocol.

### 4.5. Western Blot

HepG2 cells or adipocytes were treated with Sildenafil (1 nM to 10 uM) +/− Leucine (0.5 mM) for 2 or 4 h. Then proteins were extracted by a standard protocol and protein levels of cell extracts were measured by the BCA kit (Thermo Scientific, Pittsburgh, PA). AMPK, Phospho-AMPKα (Thr172), Sirt1, and PDE2 antibodies were obtained from Cell Signaling Technology, Inc. (Danvers, MA, USA). Equal amounts of protein (20 ug) were resolved on 10% Tris/HCL polyacrylamide gels (Criterion precast gel, Bio-Rad Laboratories, Hercules, CA, USA), transferred to nitrocellulose membranes, incubated in blocking buffer (5% NFDM in 0.1% T-TBS), and then incubated with primary antibody (1:1000 dilution), washed, and incubated with secondary horseradish peroxidase-conjugated antibody (anti-rabbit AB for AMPK and PDE2, and anti-mouse AB for EPAC1, 1:10,000 dilution). Visualization and chemiluminescent detection were conducted using a BioRad ChemiDoc instrumentation and software (Bio-Rad Laboratories, Hercules, CA). Band intensity was assessed using Image Lab 4.0 (Bio-Rad Laboratories, Hercules, CA), with correction for the background. Band intensities were normalized to stain-free blots to control for loading, as described [34,35].

### 4.6. Statistical Analysis

Data were analyzed via one-way analysis of variance and a least significant difference test was used to separate significantly different group means using GraphPad Prism version 6 (GraphPad Software, La Jolla, CA, USA, www.graphpad.com). All data are expressed as mean ± SEM.

## 5. Conclusions

In summary, we have successfully modeled the U-shaped sildenafil dose-response in vitro and utilized this model to assess potential mechanisms of this dose-response relationship. Treatment with sildenafil is accompanied by a biphasic cAMP response. PDE3 is inhibited at low concentrations of sildenafil and, thereby, induce up to a two-fold increase in cAMP, whereas, at higher concentrations, this effect is reversed due to indirect activation of PDE2. These effects on cAMP are, subsequently, reflected in changes in AMPK phosphorylation and activation, as summarized in Figure 7.

## Abbreviations

PDE	phosphodiesterase
NASH	Nonalcoholic steatohepatitis
NAFLD	Nonalcoholic fatty liver disease
eNOS	Endothelial nitric oxide synthase
NO	Nitric oxide
cGMP	Guanosine 3′,5′ cyclic monophosphate
cAMP	adenosine 3′,5′ cyclic monophosphate
EPAC	cAMP activated guanine-nucleotide exchange protein
CamKKβ	calcium/calmodulin-dependent kinase kinase β
